# Supplementary material for: Tools for the Assessment of Risk-Taking Behavior in Older Adults with Mild Dementia: A Cross-Sectional Clinical Study
Source: Brain Sci. 2023 Jun 19;13(6):967. doi: 10.3390/brainsci13060967 (PMC10296531; doi:10.3390/brainsci13060967)
Supplement: Supplementary file 1 [file brainsci-13-00967-s001.zip › brainsci-2443617-supplementary.pdf]

Supplementary materials

Box S1: Decision-making under risk

BART procedure

Balloon analog risk task (BART) is a computerized cognitive task that evaluates the risky decision-making (Lejuez et al. 2002). Participants sat comfortably in a chair in front of the screen and completed a version of the BART adapted to neuroimaging, which was implemented with E-prime 3.0. The diagram of the BART was presented in Fig. 1. Patients were instructed to inflate a virtual balloon on the screen by pressing the "pump" button to inflate a balloon or pressing the "collect" button to stop and collect money in a permanent bank, by using the response box. The probability of popping a balloon increased with each successive pump, and the balloon's reward was lost once it popped. Each insufflation was associated with 10 euros reward. They were repeatedly two possible outcomes: (1) the balloon inflated and the value increased, or (2) the balloon popped and the reward associated with it was lost. The probability of the balloon's explosion was unknown to participants.

The aim of the BART was to obtain the highest total reward by increasing the number of pumps before collection, with minimal popping of balloons. The adaptation used included 80 trials (four blocks, with 20 trials in each block) for each participant and the random probability of each balloon explosion was 2–12 pumps (the balloon could not explode at the first pump and the maximum number of pumps for each balloon was 12). The maximal potential award amount was the same for each block.

Similarly to previous studies, risk-taking behavior on the BART was measured by calculating the number of adjusted pumps, i.e., the average number of pumps on successful trials. Successful completion of the BART task involves having a high adjusted average number of pumps with a large pot's amount.

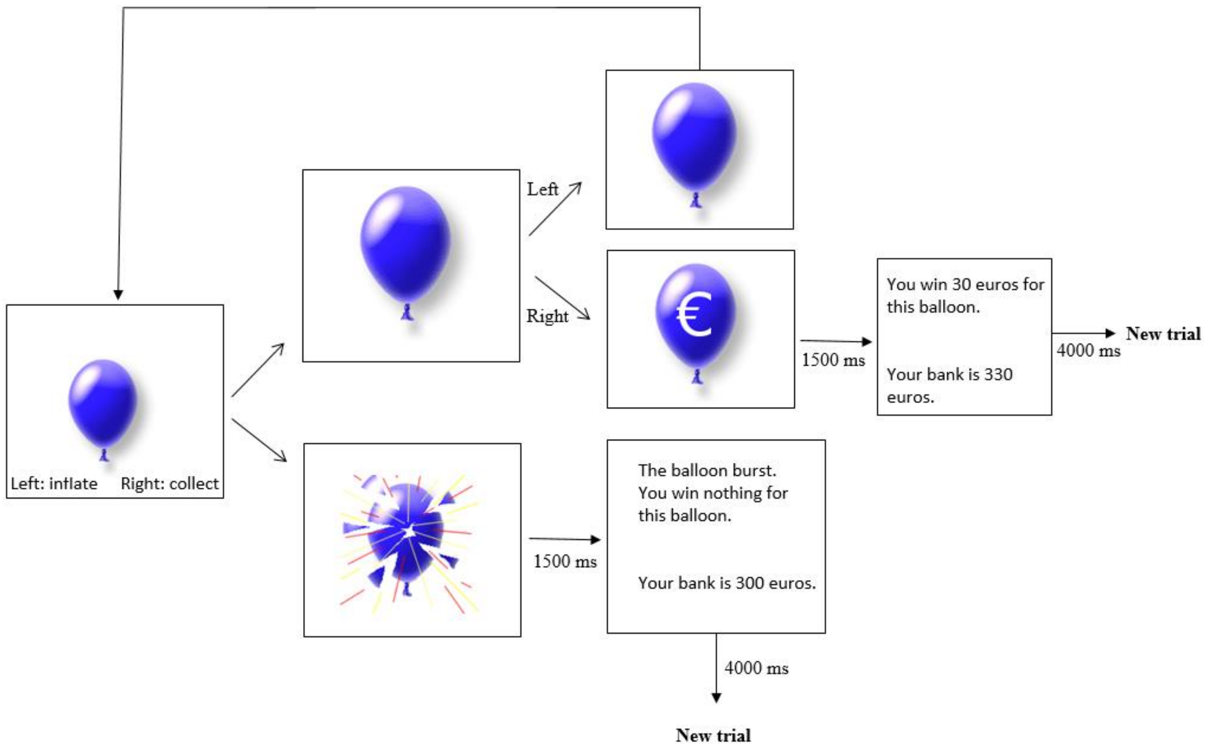

Fig. X: Schematic of the Balloon Analogue Risk Task adapted to neuroimaging. Each trial started with a small balloon. When participants chose to pump the balloon with the left button, two outcomes were possible: positive or negative feedback. Positive feedback consisted in an increase in the size of the balloon and reward amount. Participants could continue to inflate the balloon to increase the reward or put the money in a permanent bank. Negative feedback consisted in the explosion of the balloon and the loss of its reward amount.

Box S2: Decision-making under ambiguity

IGT procedure

The Iowa Gambling Task is a computerized cognitive task used to evaluate decision-making under ambiguity (Bechara et al. 1994). The original IGT involves choosing between four options, i.e., two advantageous and two disadvantageous choices. In this study, the IGT was modified from its original version (Oberg, Christie, et Tata 2011) and adapted to EEG. The simplified version of IGT was used and implemented with E-prime 2.0. A simplified version of the IGT was used to facilitate the understanding and performance of the motor aspect of the task by cognitively impaired elderly patients. In this task, participants had to choose only between two options, either a small (50 euros) bet or a large (100 euros) bet by using the response box. The gambling task included 200 bets, divided in five blocks of 40 bets. The win/loss sequence was 0.6/0.4 probability for the small bet and 0.4/0.6 probability for the large bet. The probability was randomized within each block of 40 bets. After each bet, the amount of money corresponding to the small or large bet was added or subtracted from the bank. Like in IGT, the aim of this task was to maximize the final score. Thus, the optimal strategy over the long run was to choose the small lower-risk bet.

At the beginning of the task, the bank amount was 0 euros. Participants chose between a small or a large bet, then feedback was presented for this bet for 2 s, and indicated the amount of money involved. A point in the center of the screen was presented during 3 s. A colored square appeared to indicate if the bet was won (green square) or lost (red square), and the participants had to do a new bet. The display is presented in Fig.2.

Similarly to previous studies, decision-making and risk-taking behavior on the IGT was measured by calculating the netscore, which represented the proportion of disadvantageous choices subtracted from advantageous choices. Choice 50 was advantageous, frequently associated with small rewards and a high probability of winning reward. Choice 100 was disadvantageous, rarely associated with big rewards and a large probability of losing reward. The optimal long-term strategy was to choose more frequently the 50 choice.

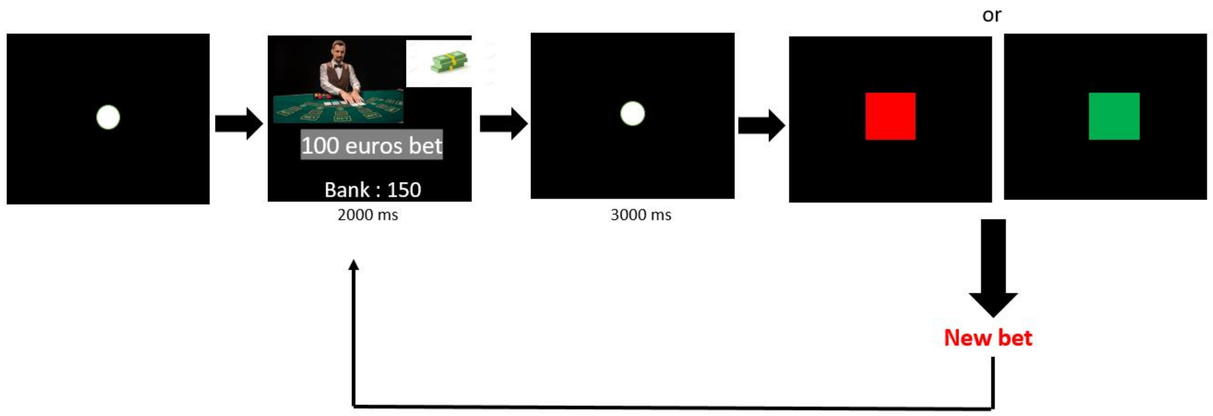

Fig.Y: Schematic of the simplified Iowa Gambling Task. First, participants choose between a small and a large bet with the response box. Then, feedback for this choice was presented during 2 s. A point in the center of the screen appeared during 3 s and the feedback was displayed until participants made a new bet.
